# Changes in phytoplankton community structure over a century in relation to environmental factors

**DOI:** 10.1093/plankt/fbac055

**Published:** 2022-10-17

**Authors:** Elisabeth Lundsør, Wenche Eikrem, Leif Christian Stige, Anette Engesmo, Sandra Gran Stadniczeñko, Bente Edvardsen

**Affiliations:** Department of Biosciences, University of Oslo, PO Box 1066 Blindern, 0316 Oslo, Norway; Norconsult AS, PO Box 626, 1303 Sandvika, Norway; Norwegian Institute for Water Research (NIVA), Gaustadalléen 21, 0349 Oslo, Norway; Natural History Museum, University of Oslo, PO Box 1172 Blindern, 0318 Oslo, Norway; Department of Biosciences, University of Oslo, PO Box 1066 Blindern, 0316 Oslo, Norway; Norwegian Veterinary Institute, PO Box 64, 1431 ås, Norway; Norwegian Institute for Water Research (NIVA), Gaustadalléen 21, 0349 Oslo, Norway; Norwegian Institute for Water Research (NIVA), Gaustadalléen 21, 0349 Oslo, Norway; Department of Biosciences, University of Oslo, PO Box 1066 Blindern, 0316 Oslo, Norway

**Keywords:** coastal phytoplankton, time series, Oslofjorden, coastal marine monitoring, eutrophication, GAM, *Emiliania huxleyi*, Skeletonema, Skagerrak, taxonomic composition

## Abstract

Changes in phytoplankton abundance and biomass during the period 1933–2020 were examined by statistical modeling using data from the Inner Oslofjorden phytoplankton database. The phytoplankton abundances increased with eutrophication from 1930s to 1970s, but with the implementation of sewage cleaning measures and a resulting reduction in nutrient releases, the phytoplankton abundance has since then decreased significantly. The onset of the seasonal blooms has started progressively later during the last 15 years, especially the spring bloom. The delayed spring bloom co-occurred with increasing temperature in winter and spring. The diatom biomass decreased more than that of dinoflagellates and other microeukaryotes. The diatom genus *Skeletonema* dominated the spring bloom and was found to be the key taxa in explaining these changes in abundance and phenology. Extensive summer blooms of the coccolithophore *Emiliania huxleyi*, which has been characteristic for the inner Oslofjorden, has also gradually decreased during the last decades, along with reducing eutrophication. Dinoflagellates have not had the same reduction in abundance as the other groups. Despite an increasing proportion of dinoflagellates compared with other taxa, there are no clear indications of increased occurrence of toxic algal blooms in inner Oslofjorden. However, the introduction of new “toxin-producing” species may cause concern.

## INTRODUCTION

Phytoplankton are the major primary producers in the oceans and produce most (ca. 98%) of the organic matter available for subsequent trophic levels of marine food webs (Charpy-Roubaud and Sournia, 1990; Field *et al.*, 1998). Phytoplankton is a key component in biogeochemical processes influencing the climate and can have important impacts on water quality, e.g. by affecting turbidity and concentration of dissolved oxygen (Litchman *et al.*, 2015). Human activities, such as eutrophication, pollution, overfishing and climate change, affect phytoplankton communities directly or indirectly and thereby affect the services they provide to ecosystems and society (Beardall *et al.*, 2009). High fluctuations in phytoplankton communities due to fast response to environmental changes make long-term changes related to natural and human-induced impacts difficult to reveal. Observations over periods of several decades are necessary to separate decadal-scale variability from long-term biological trends in marine pelagic ecosystems, but such time-series data are rare (Bindoff *et al.*, 2019).

Although phytoplankton can respond rapidly to environmental changes, shifts in light and nutrient levels through the year maintain a typical seasonal pattern in the abundance of certain taxonomic groups of the phytoplankton community (Lekve *et al.*, 2006).

In a previous study, we found a significant reduction in phytoplankton biomass (chlorophyll *a*) from the 1980s to 2018 as a result of improved wastewater treatment in the inner Oslofjorden (Lundsør *et al.*, 2020). The decrease in chlorophyll *a* correlated with a decrease in nitrogen and phosphorus concentrations in the inner Oslofjorden. A positive correlation was found between chlorophyll *a* and phosphorus during spring blooms, and chlorophyll *a* and nitrogen during autumn blooms, suggesting that the spring bloom was limited by phosphorus availability and autumn bloom by nitrogen availability. During the same period, a trend towards later spring blooms and increasing temperature in the upper water column was found (Lundsør *et al.*, 2020). Reduced nutrient concentrations could not explain all of the long-term decrease in chlorophyll *a*, and other effects of climatic changes may also be involved (Lundsør *et al.*, 2020). Increasing sea surface temperature (SST) due to climate change can result in reduced phytoplankton biomass and shifts in bloom phenology due to increased stratification and reduced nutrient availability or increased predation (Winder and Sommer, 2012). Reduced light availability due to coastal darkening (browning) may be another explanation.

In the present study, we aimed to describe the long-time changes in abundance and seasonality of the phytoplankton community and unravel changes in distribution patterns of dominant taxa, new recordings of species and changes in the abundance of taxonomic groups through almost 90 years of sampling (1933–2020).

We also aimed to understand how reduced nutrient levels and increased SST have impacted phytoplankton composition and abundance. The analyses were based on a newly compiled dataset (Lundsør, 2021), embracing comprehensive quantitative phytoplankton cell counts from the inner Oslofjorden since the 1930s and environmental data.

We addressed the following questions:

- Has the seasonal pattern of phytoplankton concentrations and taxonomic composition changed over time in the inner Oslofjorden?
- Have the phytoplankton taxa that dominate the blooms changed during the last century?
- Has the occurrence of harmful algae changed over time?
- How did changes in nutrients, salinity and temperature coincide with the seasonal and interannual changes in phytoplankton?

## MATERIAL AND METHODS

### The study area

**Fig. 1:**
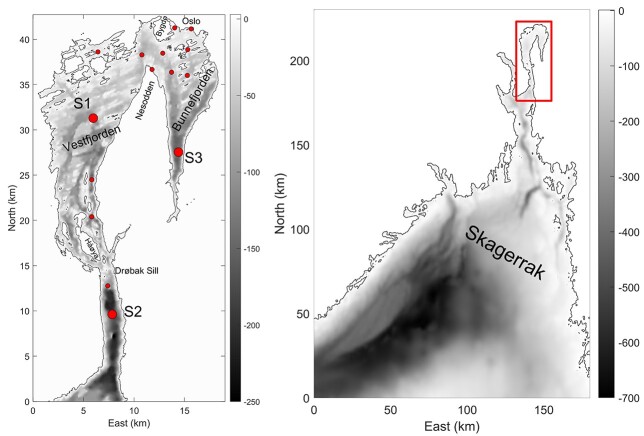
Sampling stations included in the long-term analysis. S1 (Dk1) is the main sampling station and the only station used for analysis from 1994 to 2020. S2 and S3 do also have high amount of data. Additional sampling stations are indicated with smaller dots. Exact locations can be found in (Lundsør, 2021).

The inner Oslofjorden is a sill fjord with an area of 190 km^2^. The connection to the more open outer Oslofjorden and Skagerrak in the south is through the narrow sound of Drøbaksundet, where the sill is only 19.5-m deep. Northwards of the Drøbak sill, more sills divide the fjord into several basins, such as Vestfjorden, Bærumsbassenget, Bekkelagsbassenget and Bunnefjorden. This bathymetry is a constraint to efficient deep-water renewal (Staalstrøm, 2015) that takes place in the inner basin Bunnefjorden only every 3–5 years (Baalsrud and Magnusson, 2002) (Fig. 1). Inner Oslofjorden is a relatively sheltered area with calm weather, warm summers and cold winters with typically southerly winds in the summer and northerly winds during winter (Thaulow and Faafeng, 2014).

SST and salinity varies strongly through the year in inner Oslofjorden. The stratification is mainly influenced by freshwater outflow from the rivers Glomma and Drammenselva in outer Oslofjorden, and by brackish water from the Baltic current, creating a surface layer which is much less saline than the deep Atlantic water. The horizontal water exchange in Oslofjorden takes place through estuarine circulation where low-saline surface water is flowing outwards and an underlaying high-salinity counter-current is flowing inwards the fjord (Gade, 1968). During summer, southernly winds dominate with low-saline surface waters flowing inwards and a high-salinity counter-current is flowing outwards the fjord by an inverse estuarine circulation (Gade, 1968).

The limited water exchange makes the fjord vulnerable to pollution, especially from nutrients and organic matter that may lead to high phytoplankton concentrations in surface waters and high levels of oxygen consumption in the deep-water (Staalstrøm, 2015).

Rivers, waterways and land runoff are significant contributors of bioavailable phosphate in the fjord, but the contribution from sewage plants and especially overflow runoff is also substantial. A major part of the total flux of organic substances is from sewage plants (Vogelsang, 2011).

### Sampling strategies and data

The data source for the analyses in this study is the Inner Oslofjorden phytoplankton database, which is a compilation of phytoplankton cell counts and co-occurring environmental data when available, from research and monitoring programs from 1896 to 2020 (https://doi.org/10.15468/gugesq, Lundsør, 2021). The dataset is most comprehensive for the station S1 (Dk1) in Vestfjorden but also includes data from 14 other stations in the inner Oslofjorden (Fig. 1). The sampling methods of phytoplankton have been similar over time, but the standard sample depth have varied from 0, 0–2, 4 to 5 m. During 1933–1984, phytoplankton samples were collected using Nansen bottles and from 1985 to 2020 with Niskin bottles from research vessels. In the period 2006–2018, samples were also collected with FerryBox-equipped ships of opportunity with autosamplers. A study comparing phytoplankton samples from 0–2 to 5 m at station S1 through 1.5 years in 2016–2017 found no significant differences in taxonomic composition or relative abundances between the two depths, suggesting that the upper 0–5 m mostly is mixed (Saubrekka, 2019). Phytoplankton was identified and quantified using the sedimentation method of Utermöhl (1958). Biovolume for each species/taxon was calculated from cell numbers according to HELCOM 2006 (Olenina *et al.*, 2006) and converted to biomass (μg C L^−1^) following Menden-Deuer and Lessard (Menden-Deuer and Lessard, 2000). The environmental parameters used in the analysis were SST, sea surface salinity (S), total phosphorus (P), total nitrogen (N), silicate (Si) and chlorophyll *a* (chl *a*). Chemical analyses of water samples and measurements of salinity, temperature by CTD (Conductivity Temperature Depth) were described in Lundsør *et al.* (2020).

Phytoplankton taxa were aggregated into three groups: diatoms (Diatom), dinoflagellates (Dino) and “other microeukaryotes” (Other). The group termed “other microeukaryotes” is taxonomically diverse and is dominated by unidentified phototrophic and heterotrophic flagellates and monads less than 5 μm in diameter.

Analysis of the whole time-series, from 1933 to 2020, included all 15 stations (Fig. 1), but from the period 1994 to 2020, only data from station S1 were included. Data from 2006 to 2020 were collected approximately monthly and were used in the more complex models to analyze seasonal and interannual trends in phytoplankton biomass and abundance.

### Phytoplankton data

From 1933 to 1993, only abundance data were available, but from 1994 and onwards, also biomass was calculated. The most comprehensive sampling started in 2006. For the taxonomic analysis, the names in the species list were updated to the current and valid taxonomy according to World Register of Marine Species (WoRMS Editorial Board, 2021) and Algaebase (Guiry and Guiry, 2021) to enable comparison of data through almost a century of sampling.

### Statistical analyses

A Generalized Additive Model (GAM; Wood, 2017) was used to quantify and describe the seasonal and interannual trends in the biological and physical–chemical variables. GAMs are non-parametric regression models, where the relations between the response variable and one or more explanatory variables are represented by smooth functions. This means that GAMs do not require predefined mathematical equations describing the presumed relationships, because the general shape of these relationships is captured by the smooth functions. GAMs were used as implemented in the mgcv-package version 1.8–26 (Wood, 2017) in the statistical programming environment R version 3.5.1 (R Core Team, 2017).

We first quantified the seasonal and interannual variation in biomass and/or abundance of total phytoplankton and the main phytoplankton groups. Specifically, we analyzed variation in logTotA = ln[cells/L total phytoplankton abundance], logTotC = ln[μg C/L total phytoplankton biomass]), logDiatom = ln[μg C/L diatoms], logDino = ln[μg C/L dinoflagellates] and logOther = ln[μg C/L other microeukaryotes]. Later, we used similar analysis for biomass of selected dominant taxa; *Skeletonema* spp., *Chaetoceros* spp., *Pseudo-nitzchia* spp. and *Prorocentrum* spp. Data were analyzed on ln-scale to homogenize the variance (Zuur *et al.*, 2010). For each of these alternative response variables (in equations 1–3 referred to by the generic notation X), we considered three models with different levels of complexity. The predictor variables were the day of the year (D) and year (Y).

Model M1 (Eq. 1) describes average seasonal patterns with the assumption of no trends between years

(1) \begin{equation*} {\text{X}}_{{t}}=\text{a}+\text{f}\left({\text{D}}_{{t}}\right)+{\varepsilon}_{{t}} .\end{equation*}

Here, subscript *t* refers to time, *a* is an intercept, *f* is a smooth function of *D* (a cyclic cubic spline with 15 knots, whose ends match to wrap the last day of the year to the first one in a seasonal cycle) and ε is an independent and normal distributed error term. The number of knots was fixed to compare the seasonal curves of different phytoplankton taxa in a standardized way.

Model M2 (Eq. 2) includes trends between years but has the assumption of no changes in seasonal patterns over the years

(2) \begin{equation*} {\text{X}}_{{t}}=\text{a}+\text{f}\left({\text{D}}_{{t}}\right)+\text{g}\left({\text{Y}}_{{t}}\right)+{\varepsilon}_{{t}} .\end{equation*}

Here, *a*, *f* and ε have the same interpretation as in Eq. 1 but are estimated separately from that model, and *g* is a smooth function of Y (a “thin plate regression spline”, with 5 knots, i.e. the default spline function in the mgcv package).

Model M3 (Eq. 3) includes seasonal and interannual trends and allows for possible changes in seasonal patterns over the years

(3) \begin{equation*} {\text{X}}_{{t}}=\text{a}+\text{h}\left({\text{D}}_{{t}},{\text{Y}}_{{t}}\right)+{\varepsilon}_{{t}} .\end{equation*}

Here, *h* is a two-dimensional tensor product smooth function of D and Y (a tensor product of two basis functions: a cyclic cubic spline function of D, with 15 knots, and a thin plate regression spline function of Y, with 5 knots).

We first fitted models M1, M2 and M3 to data on logTotA, logTotC, logDiatom, logDino logOther for the most consistently sampled period, 2006–2020. By comparing the explanatory power of these models, we determined how much of the variation in the data is explainable through seasonality alone (M1), through interannual trends combined with a fixed seasonal pattern (M2) and through interannual trends combined with a seasonal pattern that varies gradually between years (M3). To visualize the results of model M3 (two-dimensional model), we predicted the seasonal variation for 3 years representing the beginning, middle and end of the analyzed period (2006, 2013 and 2020). Note that short-term changes are smoothed by the model and that the model predictions for these 3 years can be interpreted as averages over several years. Model M2 was used to visualize changes over years for abundance and biomass of the three selected groups (Diatom, Dino and Other) for longer periods, specifically for 1933–2020 for abundance and 1994–2020 for biomass (limited by data availability). Model M2 was used rather than M3 due to insufficient data to reliably estimate possible changes in seasonal pattern for most of these response variables. It was thereby assumed that the seasonal pattern was constant and only abundance levels changed between years. However, to show the seasonal distribution of data, we also showed the results from model M3 as a two-dimensional contour plot of model predictions as function of year and day-of-year (as supplementary figures). Model M2 was used for analyzing long-term trends (1933–2020) in abundance of selected taxa. For the abundance data, the presence of zeros was accounted for by assuming a negative binomial distribution of the data. Here, the predictor functions model the natural logarithm of the expected value of the data.

To investigate seasonal and interannual trends in possible drivers of changes in phytoplankton biomass and abundance, we analyzed environmental variables logN = ln[μM total nitrogen], logP = ln[μM total phosphorus], logSi = ln[μM Silicate]), S(Salinity, practical salinity units, PSU) and SST (SST, SST, °C) by model M3 (Eq. 3). To facilitate comparison between trends in phytoplankton and environmental variables, all variables were analyzed by model M3, which was the model formulation that best explained the phytoplankton variations.

Permutational multivariate analysis of variance (PERMANOVA) and Distance-based redundancy analysis (dbRDA) on Hellinger-transformed data were then performed using the “adonis” and “dbrda” functions in the R “vegan” package (Oksanen *et al.*, 2019) to analyze the relationship between environmental variables and the phytoplankton biomass and at station S1.

Furthermore, we investigated how variation in nutrient concentrations was associated with variation in biomass. Model M4 (Eq. 4) estimated how phytoplankton biomass of the different phytoplankton groups (logDiatom, logDino and logOther, referred to as logX in eq. 4) correlated with phosphorus, nitrogen and silicate dependent on day-of-year

(4) \begin{align*} {\text{logX}}_{\text{t}}=&\text{b}+\text{k}\left({\text{D}}_{\text{t}}\right)+\text{m}\left({\text{D}}_{\text{t}}\right)\cdotp{\text{logPc}}_{\text{t}}+\text{n}\left({\text{D}}_{\text{t}}\right)\cdotp{\text{logNc}}_{\text{t}}\nonumber \\ &+\text{o}\left({\text{D}}_{\text{t}}\right)\cdotp{\text{logSic}}_{\text{t}}+{\varepsilon}_{\text{t}}. \end{align*}

Here, *b* is the intercept, *k*, *m*, *n* and *o* are smooth (cyclic cubic spline) functions of D, logPc is logP centred by subtracting the mean and logNc and logSic are logN and logSi, respectively, centred by subtracting the mean. The function k(Dt) describes the seasonal departures from a mean level when variations caused by nutrients are accounted for. The functions m(Dt), n(Dt) and o(Dt) give the season-dependent coefficients for, respectively, the associations of logPc, logNc and logSic with logX. These three functions thereby show how the interannual correlations between, respectively, logX and logP, logX and logN and logX and logSi change through the season. After initial analysis logSi was only used in analyzing variation in logDiatom. By analyzing the response and the nutrient variables on ln-scales, we assume linear relationships between proportional changes in nutrients and phytoplankton. A coefficient value of 1 implies that phytoplankton abundance or biomass scales proportionally with the nutrient; a coefficient value of 0 implies no relationship; while a coefficient value between 0 and 1 implies a nonlinear relationship on the original scale, with the largest changes in phytoplankton occurring when nutrient concentrations vary between low values. Coefficient values larger than 1 suggest a convex relationship that is difficult to interpret biologically. Note that this model describes statistical associations and that the model formulation does not represent a mechanistic explanation of the dynamics. The signs and magnitudes of the associations nonetheless throw light on the dynamics. We anticipate that nutrient-phytoplankton associations are negative when the nutrients are plentiful but potentially reduced by the phytoplankton, and positive when the nutrients are low and limiting phytoplankton growth.

Similarly, we investigated how variation in temperature and salinity was associated with variation in biomass of the different phytoplankton groups (model M5, Eq. 5).

(5) \begin{equation*} {\text{logX}}_{\text{t}}=\text{b}+\text{k}\left({\text{D}}_{\text{t}}\right)+\text{p}\left({\text{D}}_{\text{t}}\right)\cdotp{\text{SSTc}}_{\text{t}}+\text{q}\left({\text{D}}_{\text{t}}\right)\cdotp{\text{Sc}}_{\text{t}}+{\varepsilon}_{\text{t}} .\end{equation*}

Here, *b* is the intercept, *k*, *p* and *q* are smooth (cyclic cubic spline) functions of D, SSTc and Sc are SST and S, respectively, centred by subtracting the mean. The function k(Dt) gives the seasonal trend predicted for SSTc = 0 and Sc = 0, similar to the description of M4 above. The purpose of model M5 was to assess to which degree climate factors could explain the interannual trends in phytoplankton. Note that this model shows statistical associations that may reflect a number of direct and indirect mechanisms. For example, temperature and salinity may be indicators of water column stratification and water mass origin, which in turn influence light and nutrient availability for the phytoplankton. In addition, temperature and salinity can affect phytoplankton growth directly, as well as the activity of the competitors and predators of the different phytoplankton groups.

### Model comparison and diagnostics

Alternative models (M1, M2 and M3) for describing seasonal and interannual variation in phytoplankton variables (logTotN, logTotC, logDiatom, logDino and logOther) for 2006–2020 were compared based on the Akaike’s Information Criterion (AIC; Akaike, 1974), the generalized cross-validation score (GCV, Wood, 2017) and the percentage of deviance explained (*R*^2^). The AIC estimates the relative amount of information lost by a given model: the less information a model loses (i.e. lower AIC), the higher is the quality of that model. The GCV is a measure of leave-one-sample-out prediction error (Wood, 2017). Models with low AIC and low GCV were preferred over models with higher AIC and GCV. Model formulations that differed in AIC by less than 2 were considered as having similar statistical support (Burnham and Anderson, 2004). To assess if residuals were approximately normally distributed and had equal variance, quantile-quantile normal plots of the residuals and plots of residuals versus each covariate were visually inspected. To assess for temporal dependency, the autocorrelation function of the residuals was plotted. Significant (*P* < 0.05) positive autocorrelation would violate the assumption of independently distributed residuals and could lead to narrow confidence bands. No serious deviations from model assumptions were found (results not shown).

## RESULTS

### Abiotic conditions

**Fig. 2:**
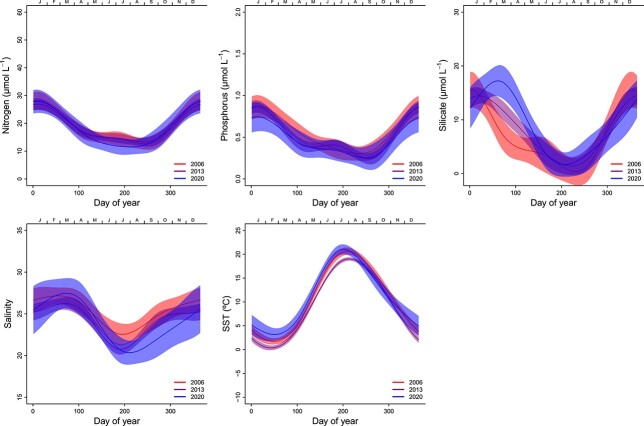
Seasonal changes in nutrients, salinity and SST from 2006 to 2020 at S1 (Dk1) in the inner Oslofjorden estimated by model M3. The solid line is the smoothed curve fitted by the GAM-model Eq. 5, and the light-colored areas represent the 95% confidence bands.

The seasonal curves for nutrient concentrations for total phosphorus (P), total nitrogen (N) and silicate (Si(OH)_4_) were similar with high concentrations during winter and lowest during summer (Fig. 2). Spring concentrations of silicate concentrations have increased since 2006, especially from 2013 to 2020 (Fig. 2). Note that while Fig. 2 only shows model predictions of seasonal variation for the first, middle and last years, the changes between years are gradual (Fig. S1). Nitrogen concentrations were slightly lower in the summer during the last period (2013–2020) compared with the first (Fig. 2), but the annual changes were not significant (Fig. S1). Phosphorus concentration did not show any significant changes from 2006 to 2020 when estimated by model M3, but the average concentrations fluctuated and were greatest in 2015–2016 and lowest in 2020 (Fig. S1).

SST was 0°C in winter and around 3–4°C during spring blooms (Fig. 2). Temperature increased to an average of around 17°C during early summer blooms, reaching its temperature peak at the end of July (day 210). The average temperature increased significantly between 2013 and 2020, especially during winter and early spring (Figs 2 and S1). Average salinity was between 25 and 27 during spring blooms and about 23 during early summer blooms (Fig. 2). Although this has varied between years, the general seasonal pattern is that the salinity in the surface layer decreases from spring towards summer, to a minimum salinity of 21 in the first half of July (around day 190) (Fig. 2). From 2006 to 2020, there has been a general decrease in the salinity of the surface waters from June to November (Fig. 2).

### Changes in yearly and seasonal patterns of phytoplankton

**Fig. 3:**
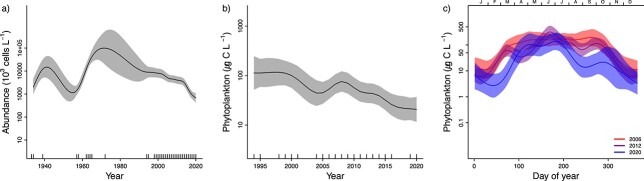
Changes in total phytoplankton biomass or abundance at station S1 in the inner Oslofjorden. (a) Yearly changes in abundance for the period 1933–2020, estimated by model M2. The solid line is the smoothed curve fitted by the GAM-model Eq. 2, and the light-colored areas represent the 95% confidence bands. (b) Yearly changes in biomass for the period 1995–2020, estimated by model M2. Seasonal patterns in biomass for the period 2006–2020 estimated by model M3. Solid line is the smoothed curve fitted by the GAM-model Eq. 5 and the light-colored areas represent the 95% confidence bands.

**Fig. 4:**
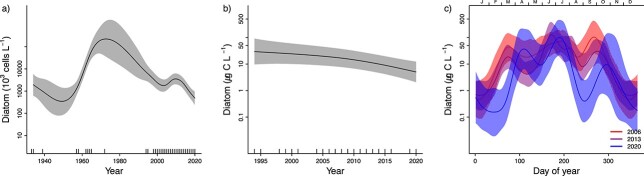
(a) Yearly changes in diatom abundance for the period 1933–2020, (b) Yearly changes in biomass for the period 1995–2020 and (c) Seasonal patterns in biomass for the period 2006–2020. For further explanation see Fig. 3.

**Fig. 5:**
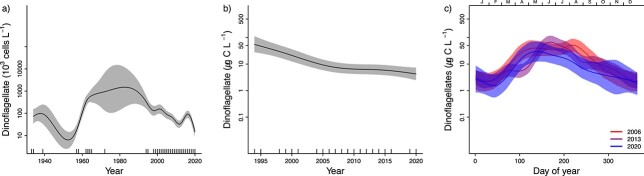
(a) Yearly changes in dinoflagellate abundance for the period 1933–2020, (b) Yearly changes in biomass for the period 1995–2020 and (c) Seasonal patterns in biomass for the period 2006–2020. For further explanation see Fig. 3.

**Fig. 6:**
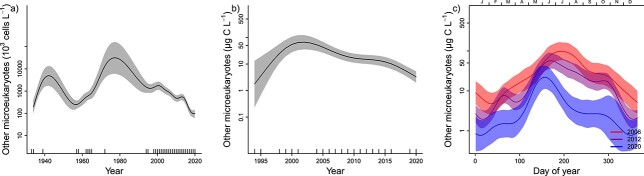
(a) Yearly changes in abundance of “other microeukaryotes” for the period 1933–2020, (b) Yearly changes in biomass for the period 1995–2020 and (c) Seasonal patterns in biomass for the period 2006–2020. For further explanation see Fig. 3.

The analyses of total phytoplankton biomass during 1994–2020 and total phytoplankton abundance during 1933–2020 show that biomass has decreased since the start of the measurements in the 1990s and abundance has decreased since the 1970s (Fig. 3a and b, Fig. S2). This pattern was similar to the pattern for diatoms (Fig. 4a and b), while dinoflagellates were recorded with the highest abundance and biomass in the period between 1970 and 2000 and have decreased thereafter (Figs 5a and b and S2). A similar pattern was found for other microeukaryotes (Figs 6a and b and S2).

Phytoplankton biomass and abundance in the inner Oslofjorden showed clear seasonality (Figs 3c and S2). The smoothed average pattern in biomass estimated by model M3 showed three annual blooms in 2020. The first peak occurred in March–April, the highest peak was in June and the third peak took place from September to October.

Changes in seasonal and yearly patterns of phytoplankton in inner Oslofjorden were studied by fitting three GAM models, with different complexity. Comparison of the three models (M1–M3) for 2006–2020 shows that the most complex model, M3, performed best for all taxonomic groups (had lowest AIC and GCV, Table 1). This indicates that both overall biomass levels and the seasonal pattern in biomass have changed since 2006. Predictions derived from the best model (M3) show that the total biomass has decreased since 2006 and the greatest monthly biomass reductions have occurred around February–March and from July to September (Fig. 3c). Furthermore, the spring and autumn peaks have moved towards later in the year. The first peak in the first half of March before 2012 had moved to second half of April by 2020 (Fig. 3c). The reduction in overall biomass since 2006 is statistically significant (*P* < 0.05 for the year-term in model M2) for total biomass (Fig. 3c) as well as for diatoms (Fig. 4c) and “other microeukaryotes” (Fig. 6c). Dinoflagellates showed a significant decline in overall biomass since 2006 and most pronounced during July and August (Fig. 5c).

**Table 1:** *GAM results of the three different seasonal models tested for each response variable (Eqs.* *1* *–* *3* *), Model M1 (Eq.* *1* *) shows average seasonal patterns with the assumption of no changes between years, Model M2 (Eq.* *2* *) includes changes through years but has the assumption of no changes in seasonal patterns over the years (i.e. effects of season and year are additive at the scale of the predictor-variable), and Model M3 (Eq.* *3* *) shows both seasonal and yearly variations, allowing for changes in seasonal patterns over the years; model performance was measured by the GCV score, AIC and percentage of deviance explained (R^2^); all models, except M2 for Prorocentrum, are significant, with P < 0.01, and the model with the lowest AIC and GCV is marked bold.*

	**M1**			**M2**			**M3**		
Response	**R2**	**AIC**	**GCV**	**R2**	**AIC**	**GCV**	**R2**	**AIC**	**GCV**
Total biomass	44.4%	1 083	1.47	52.3%	1 035	1.28	**60.8%**	**1 021**	**1.20**
Diatom	30.8%	1 476	4.77	34.5%	1 462	4.57	**46.6%**	**1 448**	**4.30**
Dino	49%	1 110	1.63	60.5%	1 033	1.29	**65.5%**	**1 027**	**1.25**
Other microeukaryotes	43.1%	725	1.39	65.4%	622	0.88	**69.8%**	**624**	**0.85**
Skeletonema	19%	979	7.04	23%	**971**	**6.77**	**34%**	971	6.88
Chaetoceros	23.1%	3 253	4.54	27.5%	3 225	4.38	**37.9%**	**3 176**	**4.12**
Pseudo-nitzschia	16.3%	976	6.94	33%	938	5.76	**40.5%**	**935**	**5.74**
Prorocentrum	**13.1%**	**861**	**4.49**	13.6%	862	4.51	17.9%	865	4.61

A change in seasonal pattern towards later spring and autumn peaks since 2006 was found for diatoms (Fig. 4c), but not for dinoflagellates (Fig. 5c) or other microeukaryotes (Fig. 5c). This result suggests that diatoms are the main driver for the observed change of the total phytoplankton biomass. Dinoflagellates show a first peak in the second half of March, and this has been constant over the 15 years analyzed (Fig. 5c). All three groups had highest biomass in June (Figs 4–6). Diatoms had the strongest decline in biomass from 2006 to 2020 in the period August–October (Fig. 4c). “Other microeukaryotes” show strongest decline from 2006 to 2020 in the period July–October (Fig. 6c).

The autumn peak in late September to mid-October was observed in the seasonal pattern for diatoms and other microeukaryotes (Figs 4c and 5c). Still, the biomass of diatoms and “other microeukaryotes” phytoplankton have decreased during this season.

The analyses of total phytoplankton biomass during 1994–2020 and total phytoplankton abundance during 1933–2020 show how biomass and abundance have decreased since the 1990s (Fig. 3a and b).

### Dominant taxa

Diatoms were overall the most abundant group both in biomass and cell numbers in our dataset, where members of the genera *Skeletonema*, *Chaetoceros* were the most dominant (Table S1). Other groups followed with coccolithophores represented by *Emiliania huxleyi* and other members of the haptophyte class Prymnesiophyceae. The most abundant dinoflagellates were members of the genus *Prorocentrum* (Table S1).

**Fig. 7:**
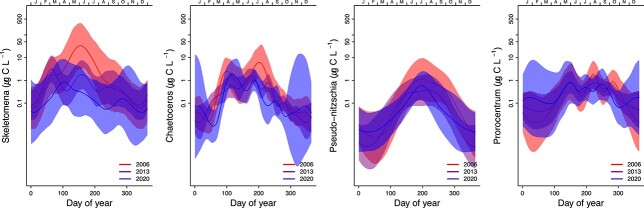
Seasonal and yearly changes in biomass in selected genera, for the period 2006–2020, estimated by model M3 at S1 (Dk1) station in the inner Oslofjorden. The solid line is the smoothed curve fitted by the GAM-model Eq. 3, and the light-colored areas represent the 95% confidence bands. Annual changes are significant for the diatoms, but not for the dinoflagellate Prorocentrum.

The typical spring bloom was dominated in terms of cell numbers and biomass by the diatom genera *Skeletonema* and *Chaetoceros*, together with the dinoflagellate genus *Prorocentrum* (Fig. 7).

**Fig. 8:**
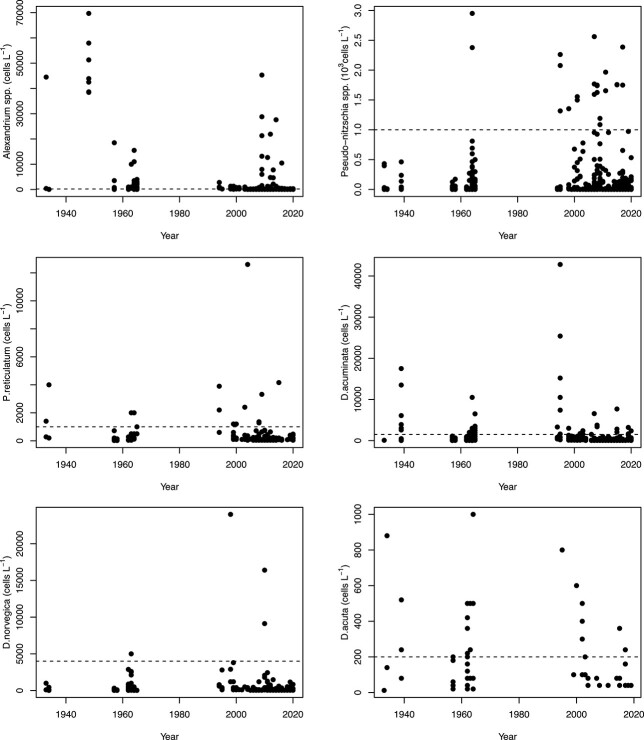
Abundance of potentially harmful algal species in inner Oslofjorden. Dotted horizontal lines represent risk levels given by the Norwegian Food Safety Authority.

Biomass of all diatom taxa investigated decreased from 2006 to 2020, especially for *Skeletonema,* as shown by reductions in AIC-value by including a year-trend in the model (comparing M2 and M1, Table 1) and by seasonal curves predicted for 2020 being generally lower than those predicted for 2006 (Figs 3a and 7a). The decrease in *Skeletonema* biomass was evident for the whole growth season, and since 2013, there has also been a shift from two spring peaks (February/March and May/June) to one (April) which coincided with the first peak of *Chaetoceros* (Fig. 7b). The autumn blooms were typically dominated by the larger centric diatoms *Dactyliosolen fragilissimus* and *Cerataulina pelagica* and the smaller members of the pennate diatom genus *Pseudo-nitzschia*, together with other microeukaryotes (data not shown). *Pseudo-nitzschia* spp. commonly reached blooming levels (>1 million cells L^−1^, Fig. 8). *Prorocentrum* had a seasonality throughout the period 2006 to 2020 without clear changes (Table 1, Fig. 7c).

Analysis of abundance since 1933 of the most common diatom taxa showed an increase towards a peak in the 1990s for *Skeletonema* and *Chaetoceros* that was followed by a decrease until 2020 (Fig. S3). There is also an indication of lower abundance of *Skeletonema* and *Kryptoperidinium* in 2020 than in the first years of the time series (1930s) (Fig. S4), while *Chaetoceros* and *Pseudo-nitzschia* have slightly greater abundances. The results further indicate that the abundances have returned to the same levels as at the beginning of the time series for the other taxa. *Emiliania* has bloomed in the summer, from June to August (Fig. S4). High abundances of *Emiliania* were recorded in 1933–1990 and then declined after 1990.

### Harmful species

The 6 most common potential toxic phytoplankton taxafound in our database (*Alexandrium* spp.*, Pseudo-nitzchia* spp.*, Protoceratium reticulatum, Dinophysis acuminata, Dinophysis norvegica* and *Dinophysis acuta*) when they occured, often exceeded the risk limits defined by the Norwegian Food Safety Authority (Fig. 8). However, further analysis of yearly trends in the period 1933–2020 (model M2) suggests a decreasing trend in abundance (Fig. S5). With the limited number of observations available these results come with a high level of uncertainty. It is worth noticing that while most of these taxa have been common in the inner Oslofjorden through the whole study period, *Alexandrium pseudogonyaulax* was first recorded in 2009. The abundance of *Pseudo-nitzschia* spp. had, as described above, the highest mean abundances in the 1990s, but the overall highest recorded cell numbers in our material were found in the 1960s (Fig. 8).

### Phytoplankton in relation to abiotic factors

The overall and the seasonal relationships between phytoplankton and abiotic factors were studied by RDA and regression analysis quantifying season-dependent correlations between abiotic variables and phytoplankton biomass.

**Fig. 9:**
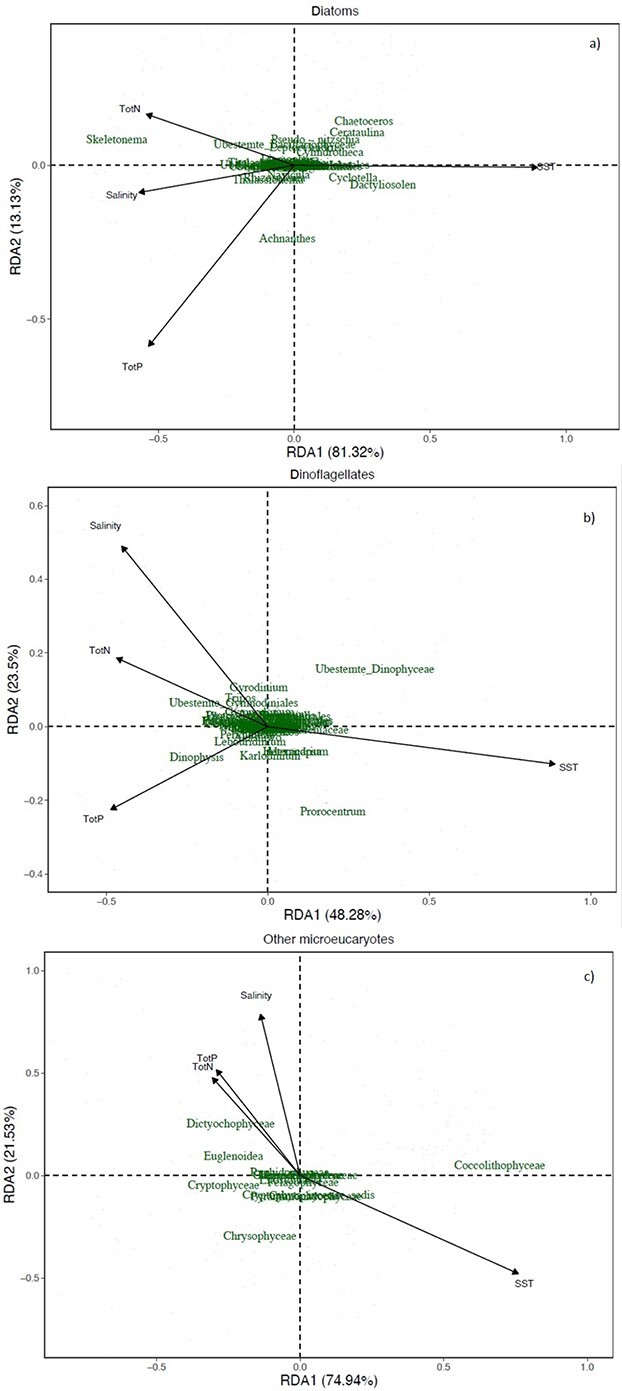
RDA ordination plot of Bray–Curtis similarities for the different studied classes at S1 (Dk1) in relation to the environmental factors.

RDA showed that the physical–chemical parameters associated differently with the class and genera studied (Fig. 9). For the class Bacillariophyceae (diatoms), *Skeletonema* positively correlated with lower temperature and higher salinity, phosphorus and nitrogen, whereas the opposite was found for *Chaetoceros, Cerataulina*, *Dactyliosolen* and *Cyclotella* (Fig. 9a). PERMANOVA analysis inferred that only temperature was a significant factor (*P* = 0.001, Table S2). However, a combination of elevated SST and salinity during spring is linked to the inflow of Atlantic seawater. Within Dinophyceae, all genera seem to be similarly associated with the studied factors (N, P, SST and salinity) with the exception of *Prorocentrum*, *Dinophysis* and some unclassified dinoflagellates (Fig. 9b), and all factors were statistically significant (*P* ≤ 0.015, Table S1). For other classes, the Prymnesiophyceae were associated with high temperatures (Fig. 9c). Temperature, salinity and nitrogen were significantly associated with the temporal variation for all other studied classes (*P* ≤ 0.01).

**Fig. 10:**
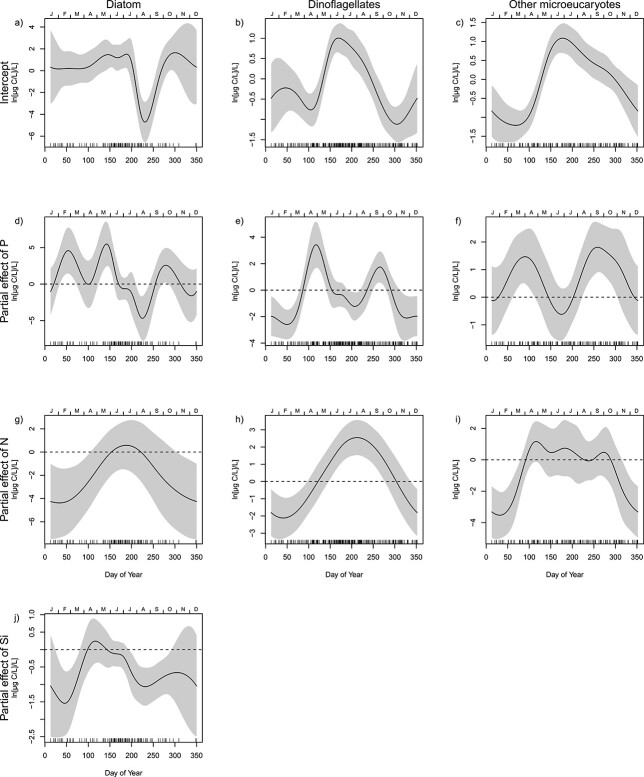
Each column shows the results of a seasonally varying coefficient model (model M4) for one phytoplankton group. The model estimates how the correlation between phytoplankton biomass and nutrients vary through the year. Each panel shows the partial effect of one variable, i.e. the change in the response by varying the given variable when all covariates are constant at zero. Panels a–c are the intercepts of each modeled group. Panels d–j show the estimated ln-scale change in biomass for one-ln-unit increase in, respectively, phosphorus (P) (d–f), nitrogen (N) (g–i) and silicate (Si) (j). The variables are centred around the long-term averages of ln(P), ln(N) and ln(Si) for a given time of year. The stippled lines show the location of zero, corresponding to no correlation between biomass and P, N or Si.

**Fig. 11:**
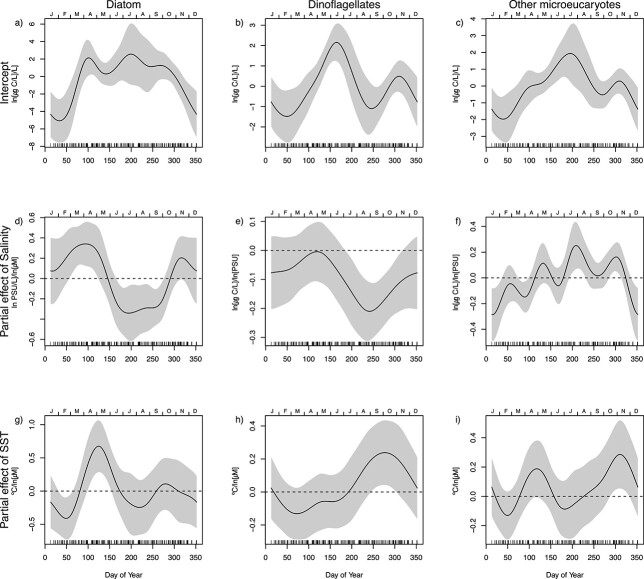
Each column shows the results of a seasonally varying coefficient model (model M4) for one phytoplankton group. The model estimates how the seasonal specific correlation between phytoplankton groups biomass and salinity vary through the year. Each panel shows the partial effect of one variable, i.e. the change in the response by varying the given variable when all covariates are constant at zero. Panels a–c are the intercepts of each modeled group. Panel d–i show the estimated ln-scale change in biomass for one-unit increases in, respectively, salinity (S) (d–f) and SST (g–i). The variables are centred around the long-term averages of S, and SST or a given time of year. The stippled lines show the location of zero, corresponding to no correlation between biomass and S and SST.

The interannual correlations between phosphorus and the biomass of all phytoplankton groups were positive both during spring and autumn (Fig. 10d, e and f). A significant positive correlation between nitrogen and biomass was only found for dinoflagellates and from May through to October (Fig. 10h). The negative correlation between silicate and diatoms during autumn corresponds to the time of year when the biomass of diatoms is increasing (Figs 2c and 10j). There was a correlation between high salinity, low temperature, and high diatom biomass during the first 3 months (Jan-Mar) of the year (Fig. 11d and g). During April and May, the correlation turned to positive between diatoms and SST, implying that high SST is associated with a delayed diatom spring bloom.

## DISCUSSION

### Seasonal succession of phytoplankton

The first reported investigation of phytoplankton in the Oslofjorden, in 1896–1897 (Hjort and Gran, 1900), showed the seasonal phenology in the inner parts of the Oslofjorden, with three distinct seasonal blooms. Increasing eutrophication led to increased phytoplankton levels (Braarud, 1969), and our study showed that the increase continued until the 1990s. Upgrading of sewage treatment was implemented gradually between 1975 and 2000, and here, we show that there was a clear and continuous reduction in phytoplankton biomass and cell numbers after the 1990s. This reduction in phytoplankton abundance corresponds to the decrease in chlorophyll *a* concentration during the same period (Lundsør *et al.*, 2020).

The distinct pattern of three yearly blooms is relatively constant throughout the whole century (Braarud and Nygaard, 1967). Winter cooling of surface waters causes unstable water masses and northerly winds mix up nutrient–rich deep waters. In early spring, the water column stratifies due to fresh and brackish water influence and heating from the sun. The first yearly bloom is set off when light availability becomes sufficient for positive gross photosynthesis due to higher solar angle and longer daylength, as well as stratification keeping phytoplankton in the well-lit upper water-mass, enhancing phytoplankton growth rate (Moschonas *et al.*, 2017). The spring bloom typically start with diatoms, followed by dinoflagellates and other micro-eukaryotes. The interannual correlation analysis suggested that especially diatoms can be limited by phosphorus during spring blooms (Fig. 10d). This correlation corresponds to the reduction in chlorophyll *a* levels in inner Oslofjorden proposed to be due to phosphate limitation during spring (Lundsør *et al.*, 2020). There has been a rise in silicate concentrations since 2006, especially during spring which may be due to increased precipitation and freshwater run off from rivers (Aksnes *et al.*, 2009) and reduced utilization from diatoms.

All available data from 1897 to 2020 show a spring bloom dominated by diatoms and dinoflagellates in March. The reduction in biomass of diatoms since 2006 corresponds to decreasing concentrations of nutrients. However, dinoflagellates did not have the same reduction as diatoms in the inner Oslofjorden. Dinoflagellates have advantageous strategies over diatoms to utilize phosphorus in phosphorus limited environments (Martínez-Soto *et al.*, 2015). They also have the ability of mixotrophy and have low requirements of silicate. Due to their capability of vertical migration, they may also exploit nutrients from deeper waters. These strategies may explain why dinoflagellates did not have the same reduction in biomass as diatoms during spring but rather exhibit a small decrease during summer and early autumn. In a previous study (Lundsør *et al.*, 2020), there were clear indications that phytoplankton biomass (as chlorophyll *a*) was nitrogen-limited during summer and autumn. The same study also suggested that the continuous reduction in nitrogen was the main controlling factor of the decreasing trend in chlorophyll *a*. Although the nitrogen reduction is no longer significant (Figs 2 and S1), the analysis of season-dependent correlations suggested that dinoflagellates are affected by nitrogen limitation during summer and autumn. Nitrogen is often found to control coastal eutrophication (Howarth and Marino, 2010).

In addition to the general decrease in phytoplankton biomass, our statistical modeling of the phytoplankton biomass showed that the onset of the first spring blooms started progressively later during the last 15 years. The delayed start of the spring blooms from 2006 to 2020 co-occurred with increasing SST in winter and spring. The analysis showed that the positive association between high SST and a delayed spring bloom was linked especially to the diatom blooms. Similar correlations between high SST and decreasing phytoplankton biomass have been found in experimental studies (Sommer and Lengfellner, 2008) and between high winter temperatures and delayed spring blooms (Borkman and Smayda, 2009). The cause behind this is still not clear. In inner Oslofjorden, the reduced diatom growth in early March may be caused by a combined effect of increased SST during winter and phosphate limited growth of diatoms during early spring, but the driving factor behind this can be another one than those that were analyzed here. Increased SST during spring was correlated to decreased mixed layer depth in the open parts of the North Sea, but not in the coastal areas (Silva *et al.*, 2021). The salinity in the surface during spring has not decreased, but an increase in SST may influence the stability of the surface layer. Increased temperatures can lead to earlier and more intensive herbivore grazing and thereby influence both the timing and the amplitude of the spring bloom (Borkman and Smayda, 2009; Lewandowska and Sommer, 2010). Following the decrease in chlorophyll in the inner Oslofjorden during the last decades the water transparency has increased, however, this has partly been counteracted by coastal water darkening (Aksnes *et al.*, 2009; Lundsør *et al.*, 2020).

### Dominant taxa

*Skeletonema* is the most common species complex in our records and among the essential contributors to phytoplankton blooms. The genera *Thalassiosira* and *Chaetoceros* are other very common and abundant diatoms in the Oslofjorden and found in records from all periods. They are also globally distributed bloom-forming diatoms, and their chain-forming life forms are highly adapted to the opportunities for fast population growth in shallow, turbulent, turbid and nutrient-enriched environments. *Skeletonema* species are fast-growing and show high plasticity in salinity and temperature tolerance and are very common throughout the coastal areas in North-Western Europe (Carstensen *et al.*, 2015). Such plasticity, as shown for *Skeletonema marinoi*, may be a result of high genetical variation within the population (Balzano *et al.*, 2011).

*Skeletonema* is the major driver of the delay in spring blooms in our study. The first peak in *Skeletonema* has weakened and been moved towards the period when also the *Chaetoceros* peak occurs, which has been rather stable in biomass through the 15 years of in-depth studies (Fig. 7b). From Oslofjorden, there are observations of *Skeletonema* in all historical records except from 1897 (Hjort and Gran, 1900; Lundsør, 2021). Only two species of *Skeletonema*, *Skeletonema pseudocostatum* and *S. marinoi*, have been identified and *S. marinoi* has been shown to be, by far, the dominant *Skeletonema* species in Oslofjorden (Valestrand, 2018). It should be noted that, *Chaetoceros* spp. includes many species of different sizes and there may have been changes within this group that would not be detected in the current study. All dominant diatom taxa have had a decrease in abundance since 1990 (Fig. S2), but only *Skeletonema* have a significant decline during the last 15 years (Fig. 7a).

In our data, *Skeletonema* was positively correlated with low temperatures together with high salinity and nutrients (Fig. 9) and showed a reduction in the abundance since 2006. This reduction is similar to the decline in total diatoms and total phytoplankton biomass, which implies that *Skeletonema* is a key taxa contributing to this change. A long-term declining trend in *Skeletonema* abundance is found in other areas where nutrient levels are decreasing, e.g. Narragansett Bay (Borkman and Smayda, 2009). The decline was found to correlate with increased water temperature and grazer abundance together with reductions in phosphorus and silicate (Borkman and Smayda, 2009). In the inner Oslofjorden, the decreasing trend of *Skeletonema* abundance and biomass was continuing, as also levels of phosphorus were decreasing. It is likely that other factors also influence this decline. For example, the decreasing *Skeletonema* abundance during spring blooms may hypothetically be caused by increasing grazing pressure due to higher winter temperatures (Wasmund *et al.*, 2017). Preferential grazing could be another reason why the genera *Thalassiosira* and *Chaetoceros* have not experienced the same decrease as *Skeletonema* the last 15 years. Unfortunately, there are no available long-term data of zooplankton phenology in the inner Oslofjorden. In our analysis, *Thalassiosira* and *Chaetoceros* are more associated with higher temperatures. The most abundant dinoflagellate during the spring blooms in inner Oslofjorden was the mixotrophic *Prorocentrum* spp. (Stoecker *et al.*, 1997) that also have been shown to perform vertical migration in Oslofjorden (Hasle, 1954). *Prorocentrum* was more correlated to higher temperatures than other dinoflagellates (Fig. 9b). It is a typically summer-blooming taxon (Horner, 2002) but is still one of the first blooming dinoflagellates. The abundance of *Prorocentrum* has had a slight decrease since the 1990s, but it is not significant. In this study, the dinoflagellate *Kryptoperidinium triquetra* (syn. *Heterocapsa triquetra*) was found in high abundance in all seasonal blooms. It typically dominated blooms in spring when freshwater discharge establishes strong salinity stratification. It was also found in autumn blooms. This pattern is found as well in other studies from North-Eastern Europe (Carstensen *et al.*, 2015)*.*

The main sampling station S1 (Dk1) is located inside the sill of Oslofjorden, with geographically reduced possibilities for water exchange with the Skagerrak. However, the phenology, including spring blooms and dominant phytoplankton taxa, are highly similar to the rest of Northern Europe (Carstensen *et al.*, 2015). One major concern for researchers and managers of the Oslofjorden has been eutrophication due to the anthropogenic release of nutrients. Already in the 1930s, very high abundance of phytoplankton was found, especially during summer. The summer plankton was often dominated by the coccolithophore *E. huxleyi* (Braarud, 1969).

*Emiliania huxleyi* is a cosmopolitan species with particularly high blooming activity in the North Sea and the Northern North Atlantic areas (Tyrrell and Merico, 2004)*.* In outer Oslofjorden, *E. huxleyi* is found all year round, with the highest relative abundances during summer and autumn (Egge *et al.*, 2015). The correlation between *E. huxleyi* and high temperatures was also clear from our analysis. Extensive *E. huxleyi* blooms were common in inner Oslofjorden with numbers as high as 12 million cells L^−1^ (Birkenes and Braarud, 1952). The abundance of *E. huxleyi* has continuously declined in the last 30 years in Oslofjorden, and no blooms above 5 million cells L^−1^ have been recorded since 2007. But blooms with levels above 1 million cells L^−1^ (Tyrrell and Merico, 2004) are still frequent during summer. Inflow from the Atlantic current of high salinity water, irradiation and temperature may be factors that promote blooms formation. There is a reduction in both cell abundance in the blooms and the frequency of summer blooms of *E. huxleyi.* Therefore, the number of blooms has gradually decreased during the last century, along with reducing eutrophication. This interpretation is consistent with statistical analysis of the relationships between seasonal plankton biomass, temperature, salinity and nutrients in the inner Oslofjorden (Lundsør *et al.*, 2020).

### Harmful algae

Generally, the inner Oslofjorden has had very few severe toxic algae events, and only one recorded incident led to human death in 1901 (Karlson *et al.*, 2021). However, a shift towards a more dinoflagellate dominated community is of concern due to the risk for more potentially toxic species. We have not found any increased frequency of harmful algal blooms during the last 15 years. However, *Alexandrium pseudogonyalax,* which produces the toxin goniodomin A (macrolide polyether) that may affect fish, shellfish and other organisms, was first recorded in 2009. The previous year it was observed by the monitoring programme in the outer Oslofjorden (Walday *et al.*, 2019). The species has since become common in the region (Karlson *et al.*, 2021).

The potential toxic diatom genus *Pseudo-nitzschia* increased in abundance from the 1960s to 2010 and since then only slightly decreased. *Pseudo-nitzschia*-species did not show a significant correlation with any of the environmental variables that were analyzed. Therefore, our results indicate no increased risk of blooms of toxic species in inner Oslofjorden. There is, however, a continued risk that additional harmful species may become established due to increasing SST.

Except for the spring blooms, there was a high degree of variation of individual species blooms, which suggests that selection of bloom-species occurs as a result of being in the right place at the right time at suitable inoculum levels (Smayda and Reynolds, 2001).

## CONCLUSION

The phytoplankton assemblages in the inner Oslofjorden have shown a striking resilience both in their major properties characterized by distinct seasonal patterns directing to some biological and functional stability. Still the trends that have been recorded for temperature, and the significant changes in the timing of the blooms signify environmental changes.

The change in the spring blooms was more correlated to temperature than the other two seasonal blooms. *Skeletonema*, which have been dominating the spring blooms, is also found to be the key taxa in explaining the changes in phenology of these blooms.

While the summer blooms of coccolithophores and diatoms found before the 1970s have been significantly reduced in abundance coinciding with reductions in the supply of nutrients, the abundance of dinoflagellates have not had the same reduction as the other groups. We suggest that this group is not as affected by the reduction in nutrient supply as the other phytoplankton groups due to different strategies of nutrient utilization and storage. Despite the greater abundance of dinoflagellates relative to other groups, there was no clear indication of an increased risk of toxic algal blooms in inner Oslofjorden; however, the introduction of new species such as *A. pseudogonyalax* is of concern.

The changes in phytoplankton phenology impact the energy available for grazers such as copepods and other zooplankton and thus affect the higher trophic levels of the food web. Delayed phytoplankton spring blooms have been found to be negatively associated with the survival of fish larvae (Platt *et al.*, 2003) and may have consequences also for the already threatened fish populations in Oslofjorden (Moland *et al.*, 2021).

## Supplementary Material

Supplementary_figure_1_fbac055

Supplementary_figure_2_fbac055

Supplementary_figure_3_fbac055

Supplementary_figure_4_fbac055

Supplementary_figure_5_fbac055

Supplementary_table_1_fbac055

Supplementary_table_2_fbac055
