# Supplementary material for: Changes in phytoplankton community structure over a century in relation to environmental factors
Source: J Plankton Res. 2022 Oct 17;44(6):854–71. doi: 10.1093/plankt/fbac055 (PMC9692196; doi:10.1093/plankt/fbac055)
Supplement: Supplementary_table_1_fbac055 [file supplementary_table_1_fbac055.docx]

Table S1: PERMANOVA results for the effect of various abiotic factors on the studied classes obtained using 999 permutations. p <0.05 are marked in bold.

|  |  | ***df*** | **SS** | **MS** | **F.Model** | **R2** | ***P*(perm)** |
| --- | --- | --- | --- | --- | --- | --- | --- |
| **Diatoms** | SST | 1 | 4.485 | 4.485 | 21.788 | 0.088 | **0.001** |
|  | Salinity | 1 | 0.307 | 0.307 | 1.493 | 0.006 | 0.155 |
|  | TotP | 1 | 0.428 | 0.428 | 2.078 | 0.008 | 0.057 |
|  | TotN | 1 | 0.349 | 0.349 | 1.694 | 0.007 | 0.113 |
|  | Residuals | 221 | 45.494 | 0.206 |  | 0.891 |  |
|  | Total | 225 | 51.062 |  |  | 1 |  |
| **Dinoflagellates** | SST | 1 | 1.610 | 1.610 | 8.570 | 0.035 | **0.001** |
|  | Salinity | 1 | 0.493 | 0.493 | 2.626 | 0.011 | **0.013** |
|  | TotP | 1 | 0.569 | 0.569 | 3.029 | 0.013 | **0.005** |
|  | TotN | 1 | 0.535 | 0.535 | 2.846 | 0.012 | **0.008** |
|  | Residuals | 225 | 42.268 | 0.188 |  | 0.929 |  |
|  | Total | 229 | 45.475 |  |  | 1 |  |
| **Other micro-eukaryotes** | SST | 1 | 2.027 | 2.027 | 17.245 | 0.069 | **0.001** |
|  | Salinity | 1 | 0.796 | 0.796 | 6.768 | 0.027 | **0.001** |
|  | TotP | 1 | 0.044 | 0.044 | 0.373 | 0.001 | 0.833 |
|  | TotN | 1 | 0.453 | 0.453 | 3.852 | 0.015 | **0.010** |
|  | Residuals | 222 | 26.099 | 0.118 |  | 0.887 |  |
|  | Total | 226 | 29.419 |  |  | 1 |  |
